# Bi-anti therapy for SARS-CoV-2 infection among mild/moderate patients to prevent coronavirus disease 2019 from progressing to severe disease

**DOI:** 10.1186/s12967-023-03965-3

**Published:** 2023-02-06

**Authors:** Jian Xu

**Affiliations:** Department of Infectious Disease, The People’s Hospital of Yubei District of Chongqing City, No.23, North Central Park Road, Yubei District, Chongqing, 401120 China

Dear editor,

Coronavirus disease 2019 (COVID-19) caused by infection with severe acute respiratory syndrome coronavirus 2 (SARS-CoV-2) is spreading around the world. The number of hospitalizations of the COVID-19 is increasing dramatically, which poses a challenge to global healthcare resources. Under the condition of limited medical resources, what can we do to reduce the hospitalization rate of patients?

After being transported to the cell surface, spike protein on the surface of SARS-CoV-2 allows the infected and healthy cells to be fused, resulting in the host cells, tissues, and organs injury [1]. Earlier studies found that the severe COVID-19 tend to have a high viral load [2]. SARS-CoV-2 infection is an initial factor to cause host damage. Therefore, it is speculated that the damage of early patients is mainly related to the direct attack of the virus on the host, antiviral therapy early might be a useful strategy for preventing disease from progressing into severe or critical cases. Drugs of direct antiviral, including nirmatrelvi/ritonavir and molnupiravir, opened a new window in the treatment of COVID-19 [3]. If these drugs of direct antiviral can be used in the outpatient department timely, the viral load of patients can be reduced as soon as possible to reduce the transmissibility, and more patients can be prevented from developing into severe or critical illness.

In addition to the direct damage caused by the virus, SARS-CoV‐2-induced host inflammatory response is a key factor in disease progression. The inflammatory response caused by SARS‐CoV‐2 is generally divided into three stages: local general inflammation stage, acute systemic inflammation stage, and chronic systemic inflammation stage of low intensity [4]. At the local general inflammation stage, SARS‐CoV‐2 infection is mainly manifested as a local inflammatory response, mainly manifested as fatigue, fever, dry cough, sore throat, and so on [5]. These symptoms reflect an underlying excessive inflammatory response to the viral infection. If the local inflammation is controlled at this stage, the disease will rarely develop into acute systemic inflammation stage where severe cases can be happened. Therefore, in addition to antiviral, anti-inflammation at the mild/moderate SARS‐CoV‐2 infection in the outpatient would prevent progression to severe illness. Non-steroidal anti-inflammatory drugs (NSAIDs) and glucocorticoids seem to be a valuable therapeutic strategy for anti-inflammatory to reduce severe COVID-19-related illness in the outpatient stage of COVID-19. Therefore, as shown in Fig. 1, we recommend “bi-anti” (antiviral and anti-inflammatory simultaneously) therapy in an outpatient setting at the early stages of SARS‐CoV‐2 infection.

**Fig. 1:**
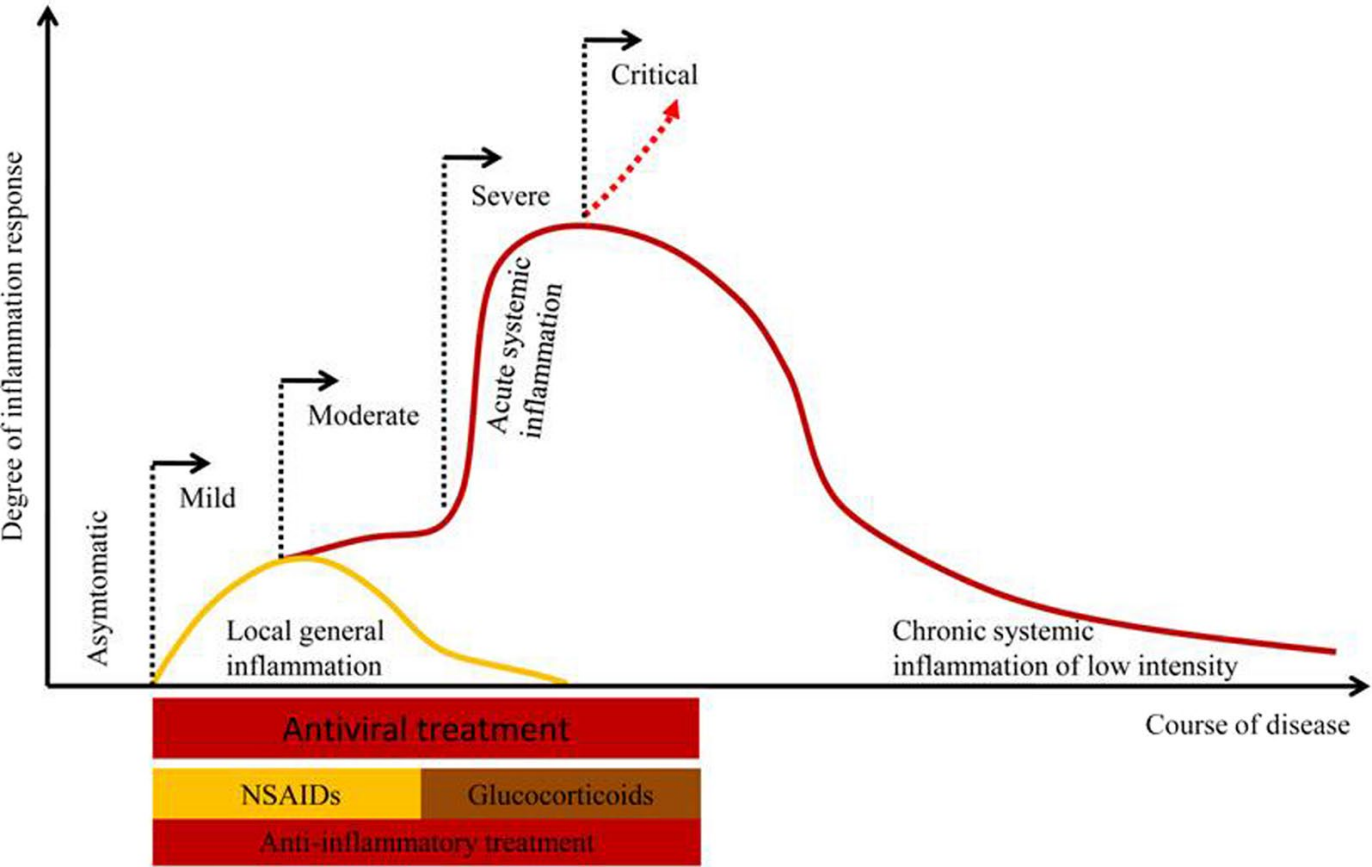
SARS‐CoV‐2 infection induced inflammatory response and “bi-anti” therapy

Overall, SARS-CoV-2 infection is only the initiating factor of the COVID-19, and the host’s immune response induced by SARS-CoV-2 is the key factor resulting in the disease progression. Therefore, we propose the concept of “bi-anti” at the mild/moderate stage of SARS-CoV-2 infection to prevent the COVID-19 from developing into severe or critical disease. However, the pathogenesis of SARS-CoV‐2 infection is complex. Early “bi-anti” therapy would provide an opportunity to intervene before infected individuals develop into severe illness. It is worthy mentioned that we mainly want to appeal to this early “bi-anti” therapy concept for mild and moderate patients, the treatment of COVID-19 should not only be limited to antiviral and anti-inflammatory, but also a comprehensive approach including anti-hypoxia, anti-coagulant, anti-infection, anti-anxiety, and anti-fibrosis therapy for severe patients (Fig. 2).

**Fig. 2:**
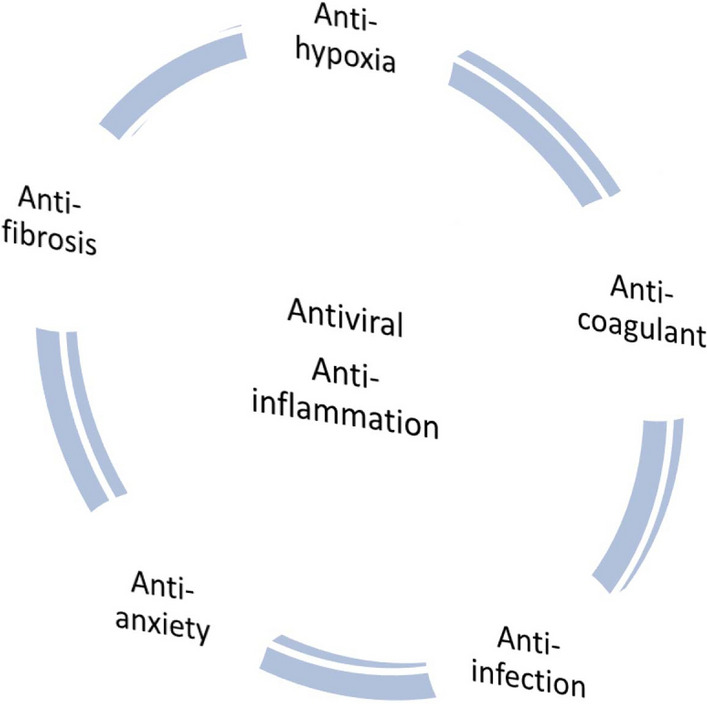
Comprehensive treatment strategies for SARS‐CoV‐2 infection

## Data Availability

Not applicable.
